# Image registration improves inter-reader agreement of objective response in CT assessment of pancreas adenocarcinoma^☆^

**DOI:** 10.1016/j.ejrad.2026.112776

**Published:** 2026-03-07

**Authors:** Jon S. Heiselman, Natally Horvat, Maria El Homsi, Burcin Agridag-Ucpinar, Onur Yildirim, Mithat Gonen, Brett L. Ecker, Eileen M. O’Reilly, Jeffrey A. Drebin, Vinod P. Balachandran, T. Peter Kingham, Kevin C. Soares, Michael I. D’Angelica, Richard K.G. Do, William R. Jarnagin, Alice C. Wei, Jayasree Chakraborty

**Affiliations:** a Department of Surgery, Memorial Sloan Kettering Cancer Center, 1275 York Ave, New York, NY 10065, USA; b Department of Biomedical Engineering, Vanderbilt University, 1225 Stevenson Center Lane, Nashville, TN, 37235, USA; c Department of Radiology, Mayo Clinic, 200 First St. SW, Rochester, MN 55905, USA; d Department of Radiology, Memorial Sloan Kettering Cancer Center, 1275 York Ave, New York, NY 10065, USA; e Department of Biostatistics, Memorial Sloan Kettering Cancer Center, 1275 York Ave, New York, NY 10065, USA; f Division of Surgical Oncology, Rutgers Cancer Institute of New Jersey, 195 Little Albany St, New Brunswick, NJ 08901, USA; g Department of Medical Oncology, Memorial Sloan Kettering Cancer Center, 1275 York Ave, New York, NY 10065, USA

**Keywords:** Pancreas cancer, Objective response, Reproducibility, Agreement, RECIST, Image registration

## Abstract

**Objectives:**

Pancreatic ductal adenocarcinoma (PDAC) exhibits ill-defined appearance on imaging during neoadjuvant therapy (NAT), which may limit consistency of dimensional measurements for objective response. This study evaluates inter-reader variability of current clinical measures of tumor size and interval response between baseline and restaging CT, and compares reader reliability with and without image registration assistance.

**Methods:**

Two senior and two junior radiologists retrospectively reviewed imaging of resectable PDAC patients enrolled in an IRB-approved Phase II clinical trial. Readers segmented lesion volumes from baseline and restaging CT and measured axial plane diameters to evaluate therapeutic response according to RECIST v1.1 and WHO criteria. Reliability of criteria index measures were compared with a computer-assisted image registration approach. Inter-reader agreements were quantified via generalized conformity index (GCI), concordance correlation coefficient (CCC), and Fleiss’ Kappa (k). Statistical analyses were performed with significance level α = 0.05.

**Results:**

Baseline and restaging imaging of 30 patients (67 ± 10y, 50% female) were evaluated. Inter-reader agreements of segmented tumor volumes at baseline/restaging were low (junior: GCI = 0.42/0.35, CCC = 0.36/0.68; senior: GCI = 0.43/0.39, CCC = 0.62/0.96; all readers: GCI = 0.43/0.38, CCC = 0.66/0.78). Agreements in percent change in tumor size differed by experience level (longest diameter: CCC = 0.26/0.77/0.59, *p* < 0.01; diameter product: CCC = 0.32/0.86/0.66, *p* < 0.001) and produced low kappa agreements (RECIST: k = 0.0/0.37/0.23, *p* < 0.01; WHO: k = 0.0/0.31/0.18, *p* = 0.01 [junior/senior/all readers]). Image registration-assisted tumor response produced higher agreements across all levels of reader experience (k = 0.82/0.80/0.81, *p* < 0.01; CCC = 0.83/0.95/0.91, *p* < 0.05 [junior/senior/all readers]).

**Conclusion:**

Substantial measurement variability exists in CT assessment of PDAC tumor response during NAT. Longitudinal image registration improves reproducibility and diminishes experience gap in radiologist assessments of objective response.

## 1. Introduction

In the treatment course of pancreatic ductal adenocarcinoma (PDAC), neoadjuvant therapy (NAT) is emerging within the standard of care to screen resection candidates for aggressive disease, control local and metastatic progression, and potentially downstage borderline disease [1,2]. Imaging assessment of PDAC response to NAT relies on interpretation of changes in vascular involvement, tumor attenuation, and tumor size that are frequently utilized as imaging markers for therapeutic response [3,4]. However, poor ability to distinguish viable tumor from fibrotic tissue, inflammation, and edema contributes to many instances where vascular invasion cannot be accurately assessed after NAT [5,6]. Additionally, intensity-based imaging markers struggle to discriminate PDAC from these pathophysiological effects. Furthermore, PDAC lesions tend to exhibit ill-defined borders relative to normal parenchyma in CT and MRI, which subsequently impedes diagnosis and complicates clinical staging [7].

Size-based measurements such as the response evaluation criteria in solid tumors (RECIST) are widely regarded to offer a robust approach for objectively measuring therapeutic response. However, due to ill-defined characteristics of PDAC lesion borders on imaging, it remains inconclusive whether size-based measures of objective response can be made with sufficient precision and reproducibility to reliably inform PDAC outcomes after NAT [2,8–12]. In baseline imaging, low inter-reader agreements of PDAC size measurements have been reported, with Dice score coefficients ranging from 0.70 to 0.73 on CT and MRI [13–15]. Yet, measurement variabilities in restaging imaging or with respect to longitudinal changes in lesion size during treatment course have not been studied beyond single-reader assessments of agreement across imaging modalities [16]. Importantly, the most widely established clinical measures of objective response, including RECIST v1.1 and World Health Organization (WHO) guidelines, were developed in the context of non-pancreatic solid tumors where it is assumed that visually apparent lesion borders result in stable measurements of percentage changes in tumor size. Ultimately, the application of these guidelines to low-contrast lesions such as PDAC may not lead to reliable assessments, despite their prevalence in clinical trials reporting and their current prominence within international recommendations for pancreas cancer management [2,17,18]. Although there is wide recognition among radiologists that RECIST and WHO criteria may lack sufficient reliability for PDAC, standard ranges for their inter-reader variabilities have not been established in the literature, and their continued use in clinical studies is perpetuated by the absence of more resilient measures for objective response that are needed to advance clinical management and personalized treatment of pancreas cancer.

Recently, longitudinal image registration was proposed for improving objective response measurement in PDAC by leveraging co-registrations between baseline and restaging imaging to reduce the accumulation of additional measurement uncertainties that would normally be introduced during repeated assessment of ill-defined lesion boundaries [19]. Image registration is an unsupervised learning approach that determines a longitudinal spatial mapping between time-interval image sequences. Image registration can thus facilitate annotation at restaging by using the learned mapping to transform geometric information such as tumor size and shape annotations between baseline and follow-up scans, essentially tracking forward the evolution of initial regions of interest identified in the baseline image. The registration process has been shown to produce highly accurate correspondences within the pancreas with average errors of 1 to 2 voxels, which enables accurate spatiotemporal mapping of intra-pancreatic anatomy throughout treatment course [19]. Image registration-derived markers for therapeutic response have thus been suggested to characterize PDAC therapeutic response with stronger correlation to survival outcomes than either RECIST or direct segmentation of PDAC tumor volumes [19]. However, it is currently unknown to what degree this unsupervised learning approach may reduce inter-reader variabilities when used by radiologists to support clinical assessment of objective response.

Beyond improving consistency within clinical assessment, enhancing measurement reliability directly impacts artificial intelligence (AI) and machine learning applications for PDAC. Radiologist annotations and reannotations often serve as ground truth data for training and validation, wherein high within-timepoint and between-timepoint inter-reader variabilities may propagate uncertainty into AI model development and ultimately limit model precision and performance. In this study, unsupervised deformable image registration is leveraged as a computational tool to assist with stabilizing longitudinal measurements, to enhance the ability for AI systems to integrate key imaging with other multi-modal information through time and across tumor, organ, and organ system scales. In the imaging context, differences in the reliability of tumor-directed measurement (the *clinical task* of lesion segmentation) compared to organ-directed measurement (the *reference task* of total pancreas segmentation) may therefore affect the downstream reliability of machine learning algorithms that build upon these guiding annotations to prognosticate outcomes or guide treatment course. Furthermore, despite the pressing need to improve clinical measurement of PDAC tumor burden, it has not yet been established which techniques (unidimensional/bidimensional diameter annotations or volumetric segmentation) may lead to the most stable within-timepoint and between-timepoint measurements. Moreover, the impact of reader experience level on inter-reader reliability has not been investigated.

To address these limitations, the objectives of this study are to characterize and assess the reliability of CT imaging assessment of PDAC at junior and senior levels of radiologist experience by comparing inter-reader variabilities among diametric and volumetric indexes for tumor burden, with and without image registration assistance to stabilize measurements of objective response after NAT. Furthermore, the impact of image registration-assisted measurements on stabilizing inter-reader reliability of objective response criteria are compared. This paper addresses a critical gap in advancing imaging measurement and annotation reproducibility, which is a necessary foundation for reliable AI systems.

## 2. Methods

### 2.1. Patient Cohort

**Fig. 1.**
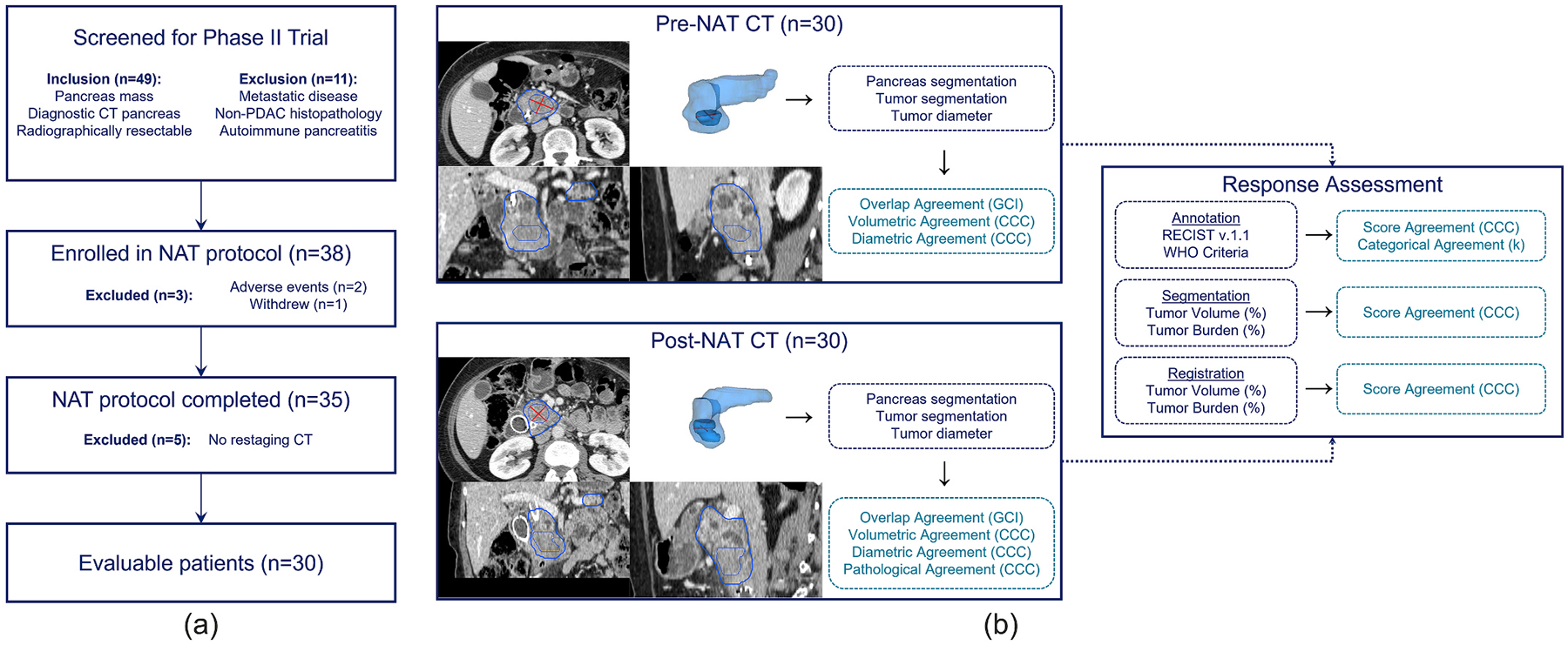
Data overview. (a) Participant selection for retrospectively analyzed study cohort. (b) Dataset assessed by junior and senior readers. Pancreas and PDAC tumor segmentations were made in pre-NAT and post-NAT CT images by 2 junior radiologists and 2 senior radiologists. Additionally, long- and short-axis diameters of PDAC lesions were annotated by each reader. Volumetric and dimensional consistencies among readers were compared at pre-NAT and post-NAT timepoints, and longitudinal measures of percent change in volume, tumor burden, and clinical objective response criteria were also characterized.

Data from a Phase II clinical trial of 38 consecutive participants prospectively enrolled between 2007–2011 to evaluate the efficacy of a NAT protocol in resectable PDAC (NCT00536874) [20] were retrospectively analyzed in this study. Informed consent was granted from all patients under the original clinical trial and retrospective analysis of these data were approved by institutional review board with waiver of Health Insurance Portability and Accountability Act authorization. Selection of the trial cohort is outlined in Fig. 1a. In all, *n* = 30 patients with histopathological confirmation of PDAC and complete baseline (pre-NAT) and restaging (post-NAT) contrast-enhanced CT imaging were included in this retrospective study for further analysis. During the clinical trial, 25 of 30 patients underwent resection upon completion of the NAT protocol. Tumor diameters associated with the resected pathological specimens were recorded for comparison with CT assessment. Survival data were recorded over 5-year follow-up. Fig. 1b shows an overview of the data workflow to assess inter-reader variabilities further described in the following sections.

### 2.2. CT Imaging

Pre-NAT and post-NAT axial plane post-contrast CT images acquired under pancreatic protocol were deidentified and exported as DICOM images. The contrast phase selected for analysis was determined based on scan availability and maximum lesion conspicuity between pancreatic or portal venous phases. Consistent contrast phases were selected between baseline and restaging images of each patient. Resolution and slice thickness ranged from 0.66×0.66×2.5 mm^3^ to 0.98×0.98×5.0 mm^3^ with equivalent axial slice spacing.

### 2.3. Manual Segmentation and Lesion Annotation

Two junior and two senior radiologists were involved in this study as independent readers. Junior readers consisted of radiology residents in training with 2 and 3 years of clinical experience, respectively, while senior readers consisted of two attending body radiologists with 11 and 7 years of experience, respectively. The presence of a solitary, pathologically confirmed PDAC tumor in each pre-NAT and post-NAT image was disclosed to each reader. However, readers were blinded to all other clinical outcomes and imaging studies of each patient.

Readers were directed to segment 3D volumes of the whole pancreas and the primary PDAC tumor in pre-NAT and post-NAT images of all patients. Additionally, readers measured the largest axial plane diameters of the tumor in pre-NAT and post-NAT images to evaluate objective response according to RECIST v1.1 via the sum of longest diameters (SLD, unidimensional) and their perpendicular diameters to evaluate WHO criteria via the sum of diameter products (SDP, bidimensional). Each reader recorded total time taken for the segmentation and annotation tasks, which were performed in ITK-SNAP (Kitware Inc., Clifton Park, NY). Segmentation and annotation times are reported in the Supplemental Digital Content (see Data, Supplemental Digital Content, Section S1: Segmentation Time). Readers were instructed on use of the software and received verification from the study team on required formats and completeness of their annotations only on the first three patients, who were included in the final analysis. All remaining segmentations and annotations were performed independently by each reader without further feedback from the study team. Numerical case identifiers and temporal sequence of the anonymized scans were not randomized so that within-patient longitudinal correlation across the NAT interval could be assessed by each reader.

### 2.4. Longitudinal Assessment of Tumor Size

For each reader, interval changes in tumor size were assessed according to percent changes in the sum of longest diameters (%Δ*SLD*) and sum of diameter products (%Δ*SDP*). Furthermore, interval changes in tumor volume were computed as the percent change (%Δ*V*) between tumor volumes in pre-NAT $\left(V_{\text{Tumor}}^{\text{Pre}}\right)$ and post-NAT $\left(V_{\text{Tumor}}^{\text{Post}}\right)$ imaging. Percent changes in tumor volume were computed from direct segmentations of the tumor volumes of each reader (%Δ*V*_*Seg*_), and from the image registration process that maps the pre-NAT tumor volume into the post-NAT image without relying on the post-NAT tumor segmentation (%Δ*V*_*Reg*_) [19]. Briefly, the image registration uses an accelerated symmetrically normalized diffeomorphic intensity-based deformable registration algorithm [21] to produce a voxel-to-voxel mapping between pre-NAT and post-NAT images as an unsupervised learning framework [19]. Using this patient-specific mapping to track evolution of treatment response at voxel scale, the tumor region of interest identified within the pre-NAT image is morphed into the post-NAT image to estimate the interval change in region size without requiring an explicit manual measurement of the lesion repeated in the post-NAT scan. Finally, as an additional comparator previously suggested to provide higher hazard ratios than %Δ*V* towards predicting survival outcomes [19], change in tumor burden Δ%*B* was also measured according to the difference in volume ratio of tumor to pancreas defined as,

(1) $$\Delta \%B=\left(\frac{V_{\text{Tumor}}^{\text{Post}}}{V_{\text{Pancreas}}^{\text{Post}}}-\frac{V_{\text{Tumor}}^{\text{Pre}}}{V_{\text{Pancreas}}^{\text{Pre}}}\right)\times 100\%,$$

where $V_{\text{Pancreas}}^{\text{Pre}}$ and $V_{\text{Pancreas}}^{\text{Post}}$ are the total pancreas volumes in pre- and post-NAT imaging. This tumor burden measure was computed directly from the volumetric segmentations prepared by each reader (Δ%*B*_*Seg*_) and with the registration-assisted workflow (Δ%*B*_*Reg*_) that mapped the baseline tumor volume into the post-NAT image to estimate $V_{\text{Tumor}}^{\text{Post}}$ in place of direct segmentation. Additional details are found in [19].

### 2.5. Statistical analysis

Inter-reader variability of pancreas segmentations, tumor segmentations, and tumor diameter annotations from each reader were compared within junior and senior groups, between reader groups, and between pre-NAT and post-NAT timepoints. Mean segmentation time was compared between junior and senior readers at both timepoints via paired *t*-test.

Inter-reader agreements in spatial overlap of segmentations were quantified using the generalized conformity index (GCI) [22], which generalizes the Jaccard index to permit comparisons across multiple readers. In the case of two readers, GCI is closely related to Dice score through the relationship Dice = 2*GCI/(1 + GCI). Overarchingly, a GCI value of zero indicates no spatial overlap of any reader segmentations and GCI of one indicates perfect spatial consistency. To assess consistency in the *clinical task*, GCI of tumor segmentation was calculated at pre-NAT and post-NAT timepoints for junior readers, senior readers, and across all radiologist readers (junior plus senior readers). GCI was similarly evaluated for the *reference task* of whole organ pancreas segmentation among radiologist readers. A comparison of GCI to CT tumor density and a comparison of inter-reader agreements of pancreas segmentation with two deep learning segmentation algorithms [23,24] are reported in the Supplemental Digital Content (see Data, Supplemental Digital Content, Section S2: Impact of CT Tumor Density and Section S3: Agreement with Deep Learning Pancreas Segmentation). Differences in GCI among readers were compared using Wilcoxon signed rank tests.

Inter-reader agreements in volume and diameter measurements at pre-NAT and post-NAT timepoints were assessed using the concordance correlation coefficient (CCC) generalized to multiple readers [25]. CCC values < 0.3 were considered poor agreement, values 0.3–0.6 as fair agreement, 0.6–0.8 as moderate agreement, and >0.8 as strong agreement [26]. Agreements between tumor diameters measured by each reader from post-NAT CT and those obtained from resected pathological specimens were also compared via CCC. Differences in CCC were statistically compared using the Z-test procedure described in [25]. Likewise, inter-reader agreements of longitudinal measures for objective response based on changes in lesion diameter, segmented volumes, and registration-assisted tracking of tumor size were assessed via CCC. Associations between longitudinal indexes for tumor response and survival outcomes were quantified by Harrell’s C-index.

Lastly, percent change in lesion diameters from each reader were stratified into CR, PR, SD, and PD groups according to RECIST v1.1 [27] and WHO criteria for objective response [28]. Additionally, %Δ*V*_*Seg*_ and %Δ*V*_*Reg*_ were compared as alternative candidates to the %Δ*SLD* index score within RECIST v1.1 using the same −30% and +20% thresholds for PR/SD and PD/SD, respectively. Categorical agreements in response groups across readers were assessed by Fleiss’ kappa. All statistical tests were performed using MATLAB 2021b (MathWorks, Natick, MA) at a significance level of α = 0.05 and were reviewed by a biostatistician.

## 3. Results

### 3.1. Patient Characteristics

The study cohort consisted of 15 male and 15 female participants with mean age of 67 ± 10 years. Patient characteristics are reported in Table 1. In this trial design, all patients were intended to receive a standardized regimen of four cycles of oxaliplatin and gemcitabine chemotherapy, followed by surgery or radiotherapy as eligible based on restaging CT. Two patients received fewer than four cycles of chemotherapy due to poor drug toleration.

### 3.2. Overlap Agreement

**Fig. 2.**
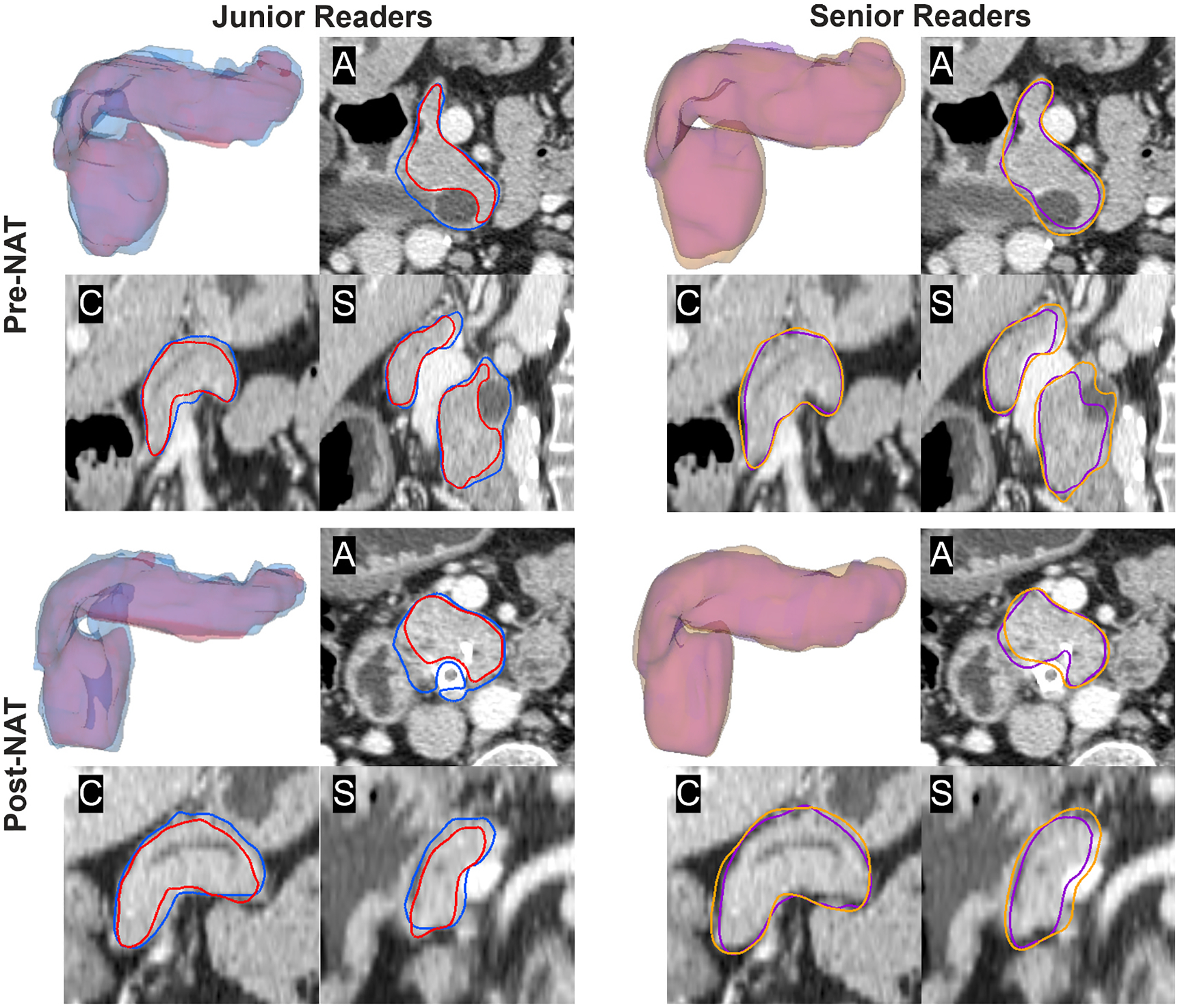
Variability of pancreas segmentations among junior readers (left) and senior readers (right) on pre-NAT and post-NAT CT in one representative patient. (A) axial slice; (C) coronal slice; (S) sagittal slice.

**Fig. 3.**
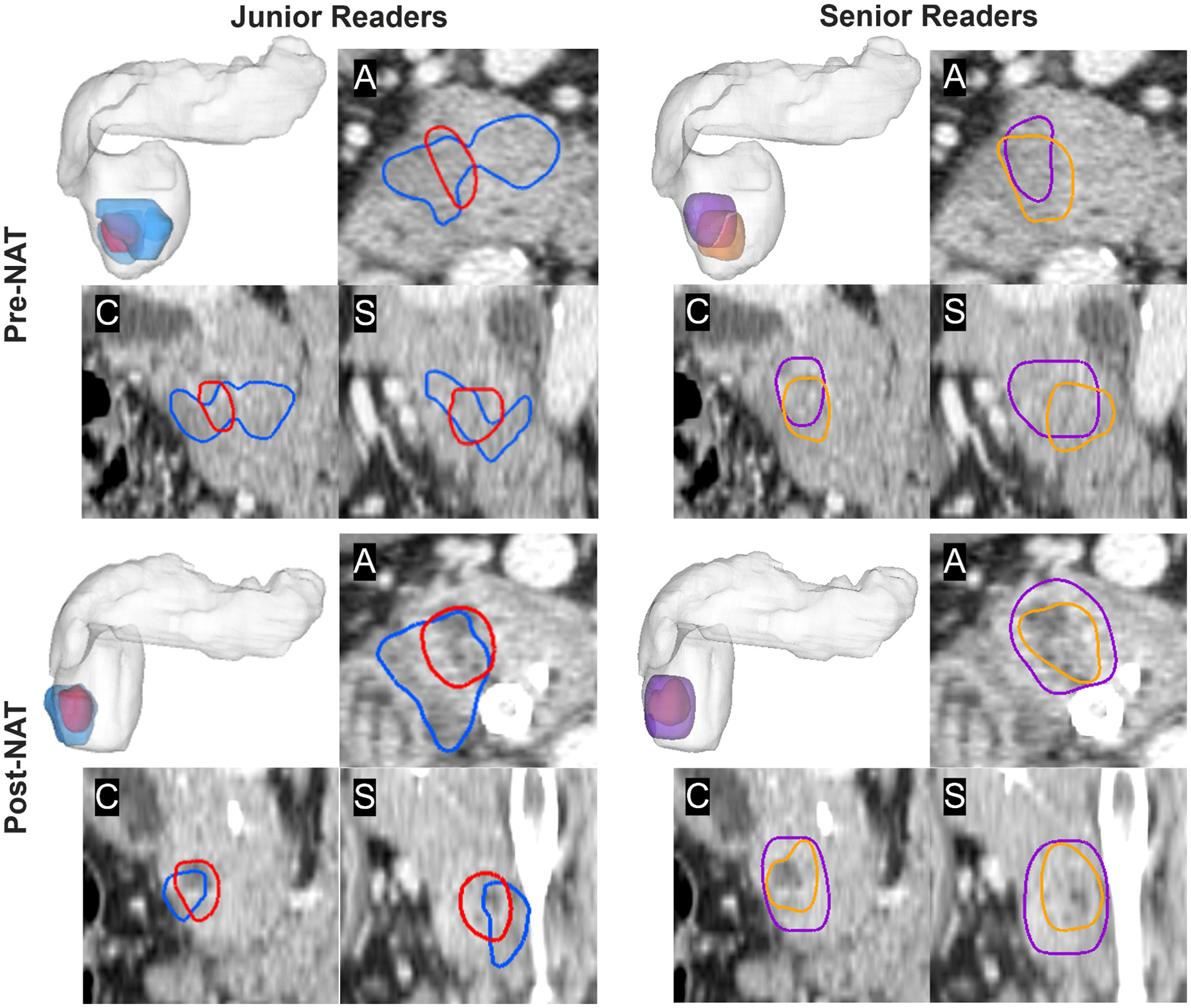
Variability of PDAC lesion segmentations among junior readers (left) and senior readers (right) on pre-NAT and post-NAT CT in one representative patient. (A) axial slice; (C) coronal slice; (S) sagittal slice.

**Fig. 4.**
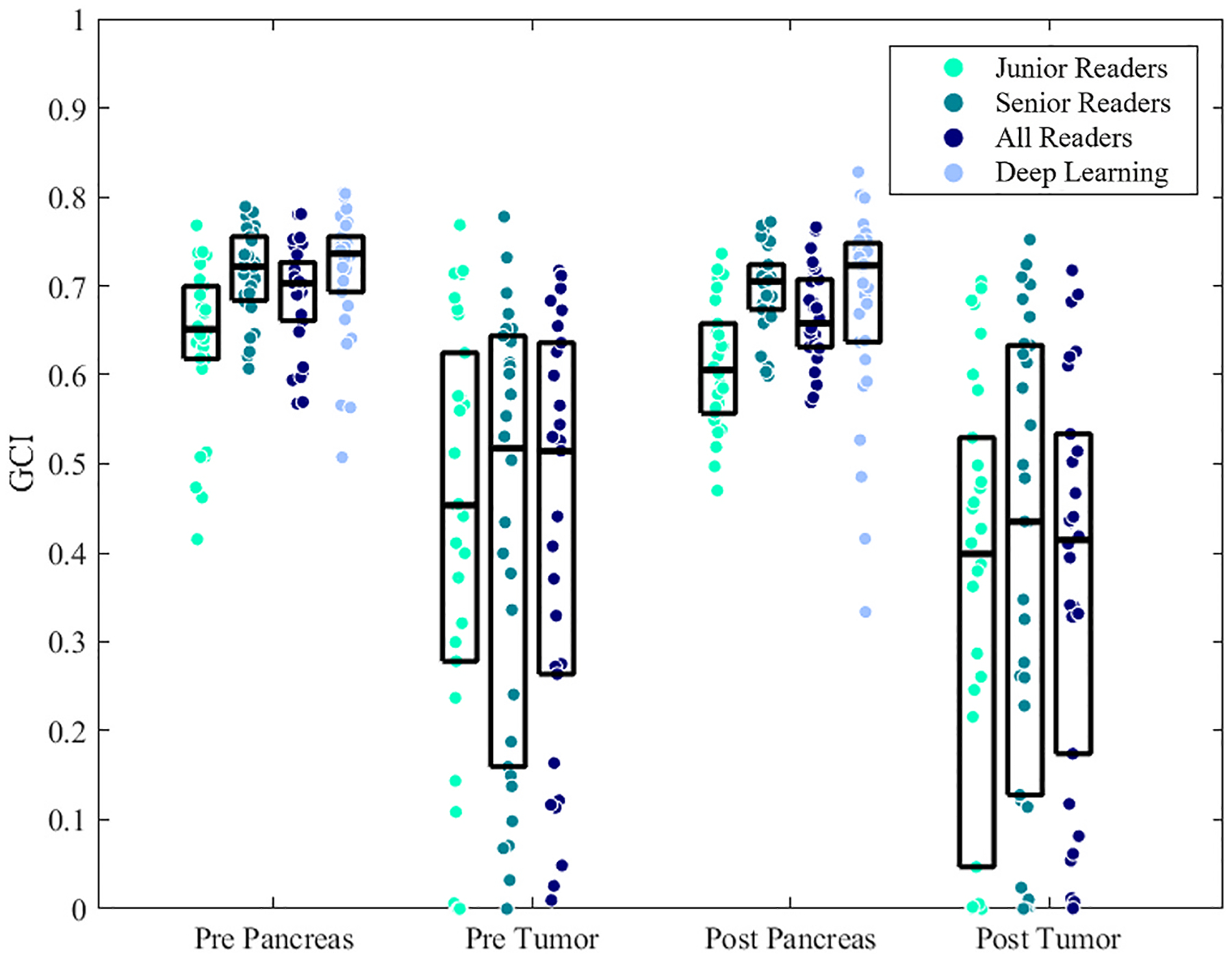
Generalized conformity index of pre-NAT and post-NAT pancreas and tumor segmentations among reader groups.

Segmentations from each reader are visualized in Fig. 2 for the *reference task* of whole organ pancreas segmentation and in Fig. 3 for the *clinical task* of PDAC tumor segmentation. Fig. 4 illustrates the GCI spatial overlap measure for pancreas and tumor segmentations among junior, senior, and all radiologists. GCI of pancreas segmentations in pre-NAT versus post-NAT imaging were 0.63 ± 0.09 and 0.61 ± 0.07 for junior readers, 0.71 ± 0.05 and 0.70 ± 0.05 for senior readers, and 0.69 ± 0.06 and 0.67 ± 0.05 for all radiologist readers. Correspondingly, GCI of PDAC tumor segmentations in pre-NAT versus post-NAT imaging were 0.42 ± 0.24 and 0.35 ± 0.25 for junior readers, 0.43 ± 0.25 and 0.39 ± 0.26 for senior readers, and 0.43 ± 0.23 and 0.38 ± 0.23 across all radiologist readers. Tumor segmentations were associated with lower GCI than whole pancreas segmentations at both junior (*p* < 0.001) and senior experience levels (*p* < 0.001). Among junior readers, GCI of tumor segmentation was lower in post-NAT than pre-NAT imaging (*p* = 0.04), whereas senior readers did not exhibit a detectable difference in GCI between tumor segmentation in pre-NAT versus post-NAT imaging (*p* = 0.34). Comparing across experience levels, GCI of pancreas segmentations was higher among senior readers than junior readers (*p* < 0.001 in both pre-NAT and post-NAT CT), although differences between GCI of tumor segmentations were not found in pre-NAT (*p* = 0.77) or post-NAT (*p* = 0.31) CT.

### 3.3. Dimensional Agreement

Table 2 reports CCC values quantifying inter-reader agreements of segmented pancreas volumes, tumor volumes, and tumor diameters, as well as their percent changes across the NAT interval as measured by junior readers, senior readers, and all readers combined.

Volumetric agreements for the pancreas segmentation *reference task* were moderate to strong among junior readers and senior readers. Senior readers demonstrated higher CCC values for $V_{\text{Pancreas}}^{\text{Pre}}$ and $V_{\text{Pancreas}}^{\text{Post}}$ than junior readers (*p* < 0.001). There was no evidence of a difference in CCC across reader groups for the percent changes in pancreas volume, with strong overall agreements exceeding 0.8.

With respect to the *clinical task* of PDAC lesion annotation, three-dimensional tumor segmentations exhibited higher CCC of $V_{\text{Tumor}}^{\text{Post}}$ measurements than $V_{\text{Tumor}}^{\text{Pre}}$ measurements at both junior (*p* = 0.04) and senior (*p* < 0.001) experience levels. Furthermore, CCC of $V_{\text{Tumor}}^{\text{Post}}$ was higher among senior than among junior readers (*p* < 0.001). When considering the interval percent change in segmented tumor volumes, CCC exhibited only fair levels of agreement among junior (CCC = 0.54) and senior (CCC = 0.52) readers. However, when measurements across all readers were pooled together, changes in tumor volume were found to lack agreement (CCC = 0.02).

Finally, agreements in two-dimensional (SDP) and one-dimensional (SLD) lesion diameters annotated by junior and senior readers were moderate-to-strong in pre-NAT and post-NAT CT. However, when considering interval percent changes, CCC across all readers was lower than CCC of baseline measurements of SDP (*p* = 0.049) and SLD (*p* = 0.041). Yet, among senior readers, CCC of percent changes in SDP and SLD did not differ from CCC of their baselines (SDP: *p* = 0.75; SLD: *p* = 0.43), which is consistent with lower precision among junior readers for assessing interval changes in lesion diameters.

### 3.4. Longitudinal Agreement of Tumor Size

**Fig. 5.**
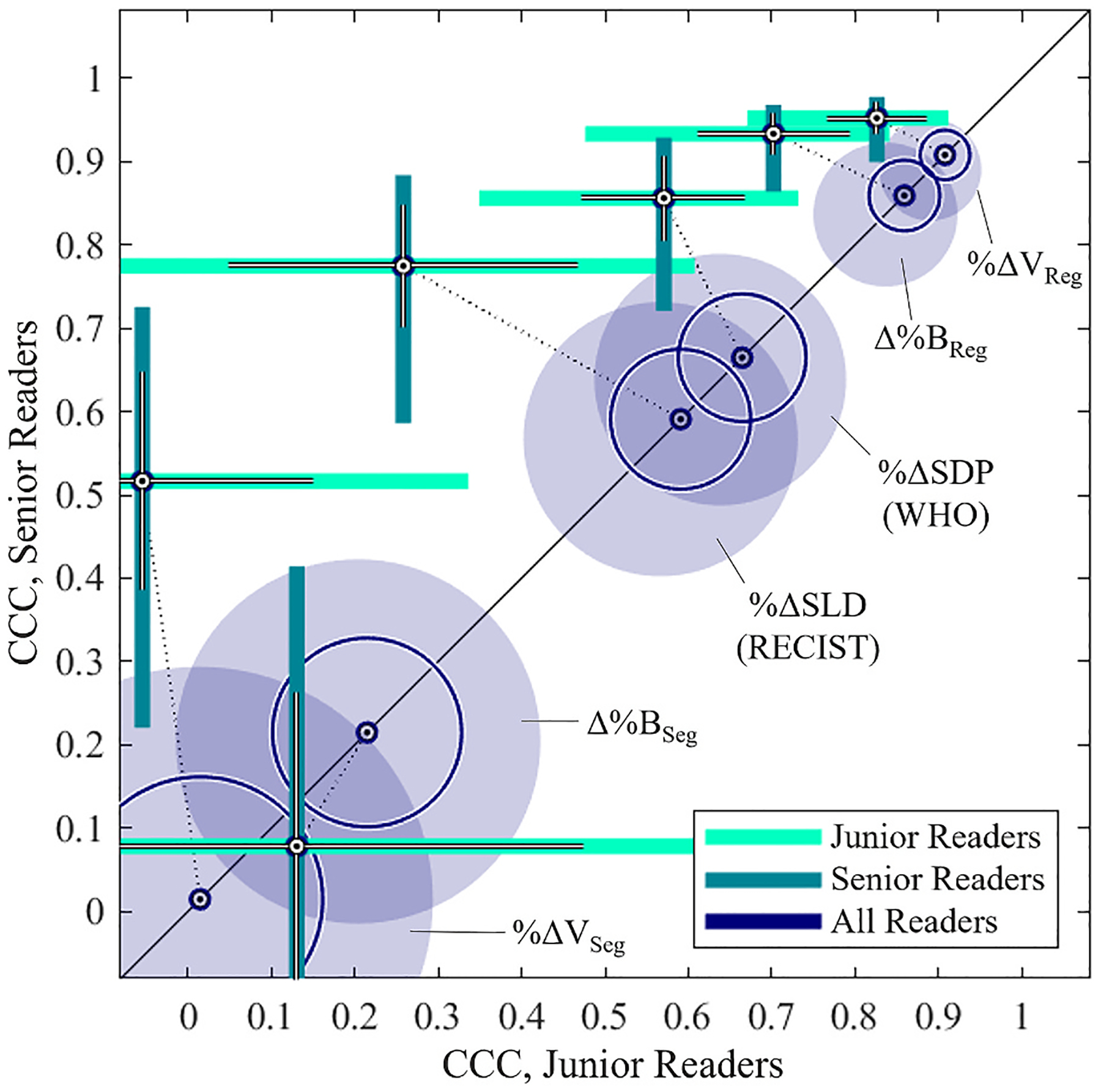
Inter-reader agreements of objective response indices. Concordance correlation coefficient of index scores for PDAC therapeutic response among junior radiologists (light green bars) and senior radiologists (dark green bars). CCC for radiologist readers of all experience levels are shown on the main diagonal (blue circles). The plot indicates the mean and standard deviation of CCC within each shaded area, which signifies the 95% confidence interval of each measurement. Dashed lines indicate the relationship between the combined CCC from all readers (blue circles) to the associated concordances among junior and senior readers (green bars) for each index score. Greater distance of the green bars from the main diagonal indicates greater effect of reader experience level on inter-reader agreement.

CCC for longitudinal measures of change in tumor size are summarized in Table 3 and illustrated in Fig. 5. Among senior readers, %Δ*SLD* exhibited higher agreements than volumetric changes measured by image segmentation (%Δ*V*_*Seg*_: *p* = 0.07; Δ%*B*_*Seg*_: *p* < 0.001), but lower agreements than volumetric changes measured with image registration assistance (%Δ*V*_*Reg*_: *p* = 0.002; Δ%*B*_*Reg*_: *p* = 0.01). Among junior readers, CCC agreement of %Δ*SLD* did not differ from agreements in volumetric changes based on manual image segmentation (%Δ*V*_*Seg*_: *p* = 0.30; Δ%*B*_*Seg*_: *p* = 0.75), but demonstrated lower agreement than the registration-assisted measures of volumetric change (%Δ*V*_*Reg*_: *p* = 0.002; Δ%*B*_*Reg*_: *p* = 0.04). Similar trends are reflected for %Δ*SDP*, where CCC of %Δ*SDP* did not differ from the CCC of %Δ*SLD* (*p* = 0.52) across all readers. Over all readers, the registration-assisted measures %Δ*V*_*Reg*_ and Δ%*B*_*Reg*_ produced higher concordances compared to measures derived from either diameter annotations (*p* < 0.05) or direct segmentation (*p* < 0.001), and exhibited less dependence on reader experience level.

### 3.5. Agreement of Objective Response Criteria

Categorization of patients into PD/SD/PR/CR groups according to RECIST v1.1 and WHO criteria by %Δ*SLD* and %Δ*SDP*, respectively, resulted in inter-reader confusion rates summarized by Fleiss’ kappa values reported in Table 4. The distribution of response category by reader and detailed confusion rates are also included (see Data, Supplemental Digital Content, Supplemental Table S2, which shows response category by reader and the resulting confusion rate). According to RECIST v1.1 and WHO criteria utilizing the changes in lesion diameters as their index scores, high inter-reader variabilities were observed when attempting to distinguish between PR and SD. Senior readers disputed the RECIST v1.1 classification in 5 of 30 patients (17%), exhibiting disagreement between PR/SD in each case, and disputed the WHO classification in 6 of 30 patients (20%), exhibiting disagreement between PR/SD in 5 cases and SD/PD in 1 case. Higher rates of disagreement were observed among junior readers in 12 of 30 patients (40%), with readers contesting PR/SD in 8 of 12 patients and SD/PD in 4 of 12 patients. When using lesion diameters for the protocol index scores, RECIST v1.1 and WHO criteria led to no agreement among junior readers (kappa values of 0.0). Senior readers exhibited higher agreements with RECIST and WHO criteria categorizations than junior readers (p < 0.01 and p < 0.05, respectively), although with modest kappa values below 0.4.

Additionally, Table 4 and Supplemental Table S2 report kappa values and confusion rates for RECIST v1.1 when utilizing %Δ*V*_*Seg*_ and %Δ*V*_*Reg*_ as alternative index scores. When RECIST v1.1 cutoffs were applied to stratify %Δ*V*_*Seg*_ scores, no difference in kappa value was found compared to RECIST v1.1 established by %Δ*SLD* (*p* = 0.88), although a substantial bias towards PR was observed. However, when RECIST v1.1 utilized % Δ*V*_*Reg*_ as an alternative index score, kappa values were higher than RECIST v1.1 established by %Δ*SLD* across all reader groups (junior readers: 0.82 vs. −0.04, *p* < 0.001; senior readers: 0.80 vs. 0.37, *p* < 0.01; all readers: 0.81 vs. 0.23, *p* < 0.001). Furthermore, the registration-assisted index measure did not cause an overall shift in the observed proportions of PD/SD/PR/CR in the study population, but dramatically reduced the rate of disagreement to only 2 of 30 patients (7%) among junior readers and 2 of 30 patients (7%) among expert readers.

### 3.6. Agreement with Pathology

**Fig. 6.**
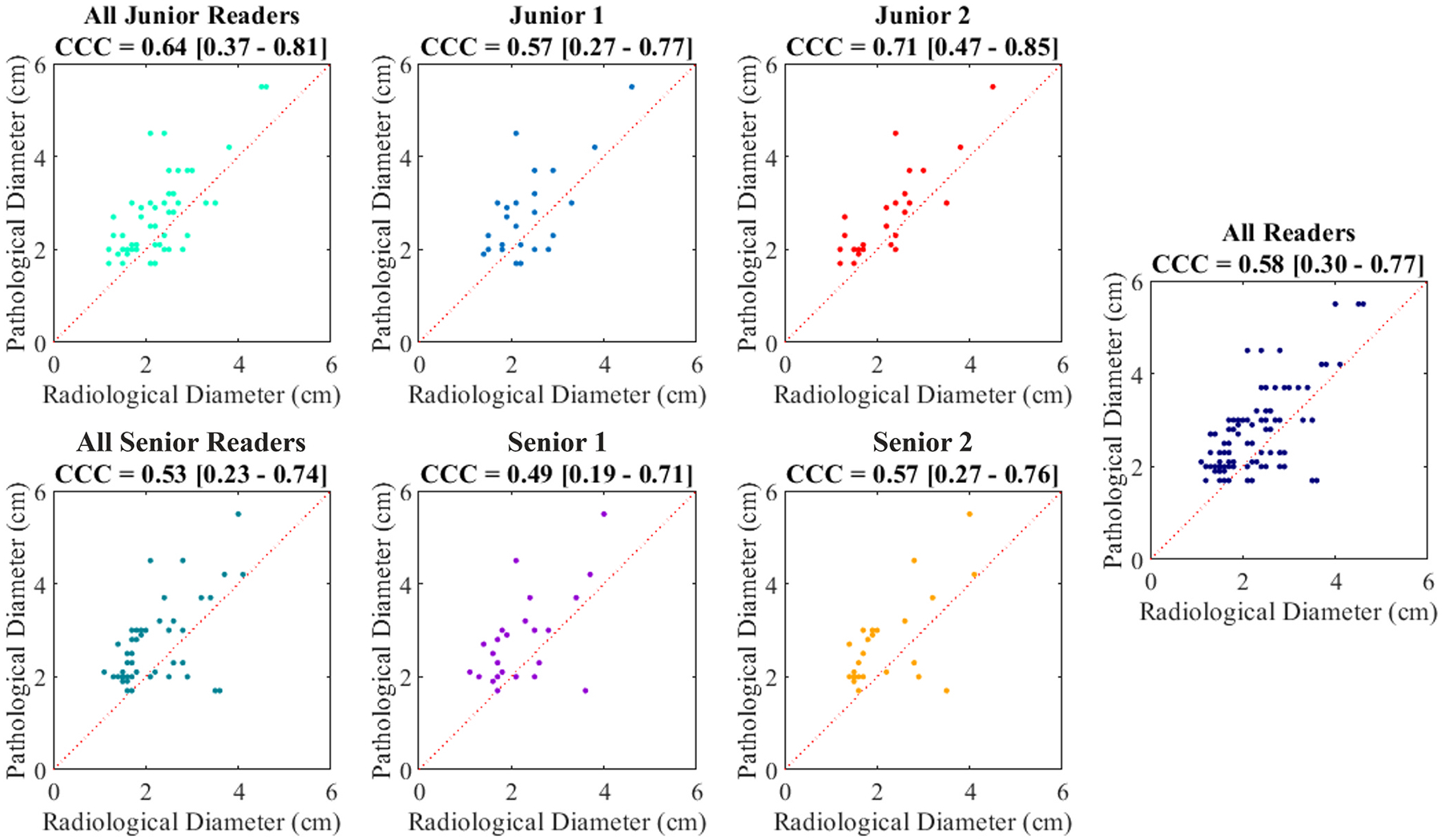
Agreement of post-NAT CT tumor diameter from each reader with pathological diameter from resected tumor specimen. CCC values are reported with 95% confidence intervals.

Longest diameters of lesions annotated by each rater in post-NAT CT were compared to ground truth post-resection tumor diameters from pathology. Correlation plots for each reader are shown in Fig. 6. CCC of junior reader agreements to pathology was 0.64 (95% CI: 0.37–0.81), which did not significantly differ from CCC of senior reader agreements to pathology of 0.53 (95% CI: 0.23–0.74) (p = 0.51). On average across all readers, radiological annotations tended to underestimate lesion diameters from pathology by 0.49 ± 0.72 cm (95% CI: 0.35–0.63 cm; *p* < 0.001).

### 3.7. Association with Survival Outcomes

**Fig. 7.**
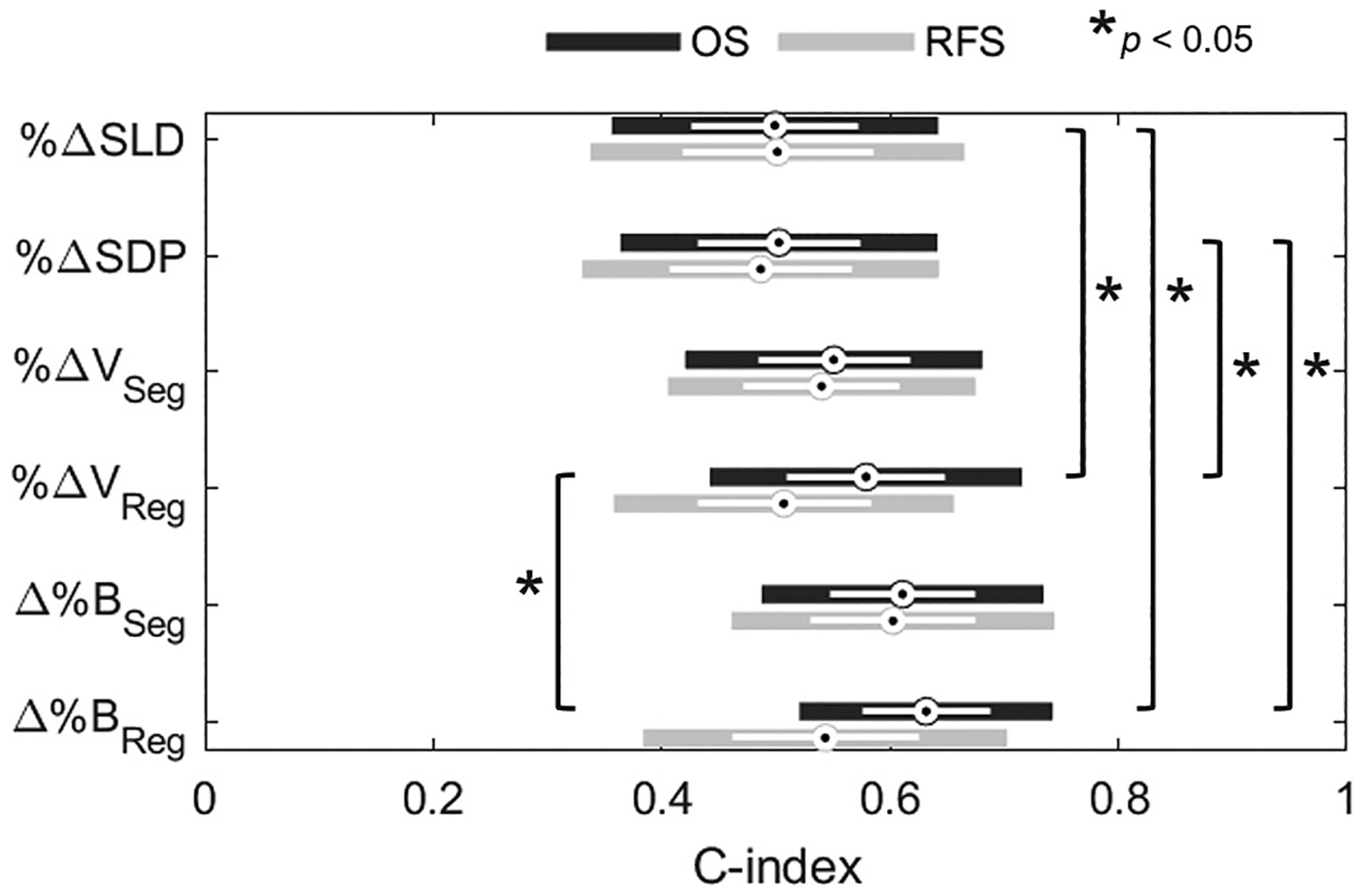
Strength of association (Harrell’s C-index) of longitudinal measures for PDAC tumor response with respect to OS and RFS. White bars indicate mean and standard deviation surrounded by solid region as 95% confidence interval. Image registration-assisted indexes exhibit stronger correlation to OS than the diametric indexes utilized in RECIST and WHO criteria for objective response.

Overall survival (OS) and recurrence-free survival (RFS) outcomes were collected over 5-year follow-up. Associations between imaging marker scores for therapeutic response and survival outcomes were quantified with Harrell’s C-index. Fig. 7 shows the mean, standard deviation, and 95% confidence intervals of C-index to each univariate measure of tumor response (also see Data, Supplemental Digital Content, Supplemental Table S3, which reports numerical ranges). Only Δ% *B*_*Reg*_ was found to produce C-index above 0.6 and provided the strongest association with overall survival compared to all other markers, whereas no significant differences among associations with RFS were observed. Registration-assisted markers of therapeutic response (Δ%*B*_*Reg*_ and %Δ*V*_*Reg*_) were found to continuously index OS more accurately than % Δ*SLD* and %Δ*SDP* utilized in RECIST v1.1 and WHO criteria, respectively (Δ%*B*_*Reg*_: 0.63 and %Δ*V*_*Reg*_: 0.58 vs. %Δ*SLD*: 0.50 and %Δ*SDP*: 0.50; all *p* < 0.05).

## 4. Discussion

Precise delineation of PDAC is a challenging imaging task due to ill-defined borders and isoattenuating characteristics of these lesions. This study reports a comprehensive analysis of inter-reader agreement of PDAC lesion segmentation and size annotation in pre-NAT and post-NAT CT relative to whole pancreas segmentation among junior and senior radiologists. Furthermore, inter-reader variabilities of longitudinal assessments for therapeutic response based on diameter annotation, volumetric image segmentation, and image registration approaches were evaluated. Our main findings are that (1) overlap agreement of PDAC lesion annotations was highly variable at junior and senior levels of clinical experience, with average GCI below 0.5 and per-patient reader agreement yielding GCI values between 0 and 0.8, (2) inter-reader agreements of longitudinal measures for tumor response based on diameter annotations and volumetric segmentations were moderate to poor, with CCC below 0.7 when comparing across all readers, and (3) image registration-assisted longitudinal assessments were associated with dramatically higher inter-reader agreements, with CCC scores of approximately 0.9.

PDAC tumor segmentations were associated with low spatial agreement and poor longitudinal consistency in percent changes of diametric and volumetric measures, likely due to differences in the ability to precisely estimate lesion borders across imaging timepoints considering the ill-defined nature of PDAC lesions. Treatment-related effects including encapsulating fibrosis and pancreatitis [29,30] may alter enhancement patterns around the tumor and further deteriorate spatial consistency of tumor segmentations across readers and through the NAT interval. We found that reader assessments of tumor diameter in post-NAT CT underapproximated lesion size measured from resected pathology specimens by approximately 0.5 cm, which corroborates other published reports [31–33] that found post-NAT CT diameters to underestimate histopathological measurements of viable tumor by 0.4–0.7 cm on average. Biological effects such as perineural tropism [34] that are more evident in histopathological imaging and treatment-related fibrosis, which can obscure residual viable tumor on imaging, likely contribute to underapproximation of lesion size in restaging CT. The unsupervised deformable image registration approach, which maintains spatially consistent regions of interest from baseline through restaging imaging, may become an assistive decision-support tool for radiologists to track bulk effects of pathologic-scale changes more reliably than repeated size annotations in low-visibility solid tumors.

In this study, substantial inter-reader variabilities were observed in RECIST and WHO criteria, which contextualizes the reliability of current objective response measures for informing treatment decisions or serving as surrogate endpoints in PDAC drug therapy trials. Even among senior readers, the kappa values < 0.4 observed in this study are lower than prior reports for PDAC [16,35], suggesting that reliability of these commonly accepted objective response criteria likely depends on institution and imaging characteristics of the patient cohort. However, image registration assistance produced an index for RECIST that led to kappa agreements > 0.8 across both levels of reader experience. These machine-augmented measures of objective response were found to produce higher CCC inter-reader agreements than volumetric measurements based on image segmentation as well as current diameter-based indexes used within RECIST and WHO guidelines. Moreover, differences in CCC between junior and senior experience levels were smaller for registration-assisted measures than for diametric or volume segmentation approaches. In further support, these registration-assisted measures for therapeutic response also offered better continuous association with survival outcomes. Overall, these results suggest that longitudinal image registration assistance offers an opportunity for radiologists to improve reproducibility of longitudinal image annotations and moderate the impact of reader skill on measurement of PDAC objective response.

## 5. Conclusion

Personalized medicine relies on dependable measurements to serve as the foundation for treatment planning, monitoring, and customization. This work shows that measurements of PDAC tumor extent are associated with low inter-reader agreements across radiologist experience levels in baseline imaging, follow-up imaging, and interval percent changes in tumor size. However, longitudinal image registration can be used to propagate initial size-based annotations from baseline into restaging imaging to enable more robust measurements of therapeutic response and to serve as a decision-support tool that radiologists can use to improve consistency of their measurements. Registration-assisted measurements of objective response were found to achieve better inter-reader agreement in response categorization and better association with survival outcomes than measurements made without image registration assistance, while reducing dependence of objective response assessment on the level of reader experience, altogether enhancing their utility within clinical and AI workflows.

## Supplementary Material

1

## Figures and Tables

**Table 1:** Characteristics of patient cohort (N = 30).

Variable	N (%)
Sex (male), n (%)	15 (50%)
Age (y), median (range)	69 (40–81)
Tumor Location, n (%)	
Head	23 (76%)
Neck	2 (7%)
Body	2 (7%)
Tail	3 (10%)
NAT Interval (days), median (range)	42 (14–49)
2 cycles	1 (3%)
3 cycles	1 (3%)
4 cycles	28 (94%)
Interval from end of NAT to restaging CT (days), median (range)	10.5 (3–21)
Additional treatment after NAT, n (%)	
Surgical resection	22 (83%)
Surgical resection + Adjuvant radiotherapy	3 (10%)
None	5 (17%)
Interval from restaging CT to surgery (days), median (range)	16 (6–27)
Pathology Tumor Grade, n (%)	
Well differentiated	1 (3%)
Moderately differentiated	20 (67%)
Poorly differentiated	4 (13%)
Unknown	5 (17%)
Pathology Tumor Response (Evans’ Criteria), n (%)	
Grade I	6 (7%)
Grade IIa	12 (40%)
Grade IIb	4 (13%)
Grade III	1 (3%)
Grade IV	0 (0%)
Unknown	7 (23%)

**Table 2:** Concordance correlation coefficient with 95% confidence intervals for agreement of pancreas and PDAC tumor segmentation volumes and lesion diameters.

Quantity	Junior Readers	Senior Readers	All Readers	Z-test Junior/Senior
$V_{\text{Pancreas}}^{\text{Pre}}$	0.54 [0.37–0.67]	0.88 [0.78–0.94]	0.66 [0.51–0.77]	*p* < 0.001
$V_{\text{Pancreas}}^{\text{Post}}$	0.64 [0.48–0.75]	0.91 [0.84–0.95]	0.75 [0.63–0.84]	*p* < 0.001
%**Δ*V***_***Pancreas***_	**0.85 [0.72–0.93]**	**0.88 [0.76–0.94]**	**0.80 [0.67–0.89]**	***p* = 0.70**
$V_{\text{Tumor}}^{\text{Pre}}$	0.36 [0.08–0.59]	0.62 [0.36–0.79]	0.66 [0.48–0.79]	*p* = 0.15
$V_{\text{Tumor}}^{\text{Post}}$	0.68 [0.47–0.82]	0.96 [0.93–0.98]	0.78 [0.64–0.87]	*p* < 0.001
%**Δ*V***_***Tumor***_	**0.54 [0.25–0.74]**	**0.52 [0.22–0.73]**	**0.02 [−0.27–0.29]**	***p* = 0.91**
*SDP* ^*Pre*^	0.74 [0.53–0.86]	0.83 [0.68–0.91]	0.84 [0.72–0.91]	*p* = 0.35
*SDP* ^*Post*^	0.82 [0.66–0.91]	0.87 [0.74–0.93]	0.87 [0.76–0.93]	*p* = 0.52
%**Δ*SDP***	**0.32 [−0.05–0.62]**	**0.86 [0.72–0.93]**	**0.66 [0.49–0.79]**	***p* < 0.001**
*SLD* ^*Pre*^	0.61 [0.34–0.78]	0.85 [0.70–0.92]	0.80 [0.66–0.88]	*p* = 0.04
*SLD* ^*Post*^	0.74 [0.53–0.86]	0.87 [0.74–0.94]	0.84 [0.71–0.91]	*p* = 0.15
%**Δ*SLD***	**0.26 [−0.17–0.61]**	**0.77 [0.59–0.88]**	**0.59 [0.40–0.73]**	***p* = 0.008**

SDP: sum of diameter products; SLD: sum of longest diameters.

**Table 3:** Concordance correlation coefficient with 95% confidence intervals for agreement of annotation-, segmentation-, and registration-assisted measures of change in tumor size across NAT.

Quantity	Junior Readers	Senior Readers	All Readers	Z-test Junior/Senior
*Annotation*				
%Δ*SLD*	0.26 [−0.17–0.61]	0.77 [0.59–0.88]	**0.59 [0.40–0.73]**	*p* = 0.008
%Δ*SDP*	0.32 [−0.05–0.62]	0.86 [0.72–0.93]	**0.66 [0.49–0.79]**	*p* < 0.001
*Segmentation*				
%Δ*V*_*Seg*_	0.54 [0.25–0.74]	0.52 [0.22–0.73]	**0.02 [−0.27–0.29]**	*p* = 0.91
Δ%*B*_*Seg*_	0.13 [−0.50–0.67]	0.08 [−0.28–0.41]	**0.22 [−0.01–0.42]**	*p* = 0.89
*Registration*				
%Δ*V*_*Reg*_	0.83 [0.67–0.91]	0.95 [0.90–0.98]	**0.91 [0.83–0.95]**	*p* = 0.01
Δ%*B*_*Reg*_	0.70 [0.48–0.84]	0.93 [0.86–0.97]	**0.86 [0.75–0.92]**	*p* = 0.002

%Δ*SLD*: percent change in sum of longest diameters; %Δ*SDP*: percent change in sum of diameter products; %Δ*V*_*Seg*_: percent change in segmented tumor volume; Δ%*B*_*Seg*_: change in tumor burden ratio via image segmentation; %Δ*V*_*Reg*_: percent change in registered tumor volume; Δ%*B*_*Reg*_: change in tumor burden ratio via image registration.

**Table 4:** Fleiss’ kappa values and 95% confidence intervals for each index measure according to cutoff scores of RECIST v1.1 and WHO criteria for progressive disease, stable disease, partial response, and complete response.

Criteria (Index Score)	Junior Readers	Senior Readers	All Readers
RECIST v1.1 (%Δ*SLD*)	−0.04 [−0.11–0.03]	0.37 [0.29–0.44]	**0.23 [0.20–0.26]**
WHO Criteria (%Δ*SDP*)	−0.04 [−0.11–0.03]	0.31 [0.23–0.38]	**0.18 [0.15–0.21]**
RECIST v1.1 (%Δ*V*_*Seg*_)	−0.16 [−0.24–0.07]	0.45 [0.37–0.53]	**0.24 [0.21–0.28]**
RECIST v1.1 (%Δ*V*_*Reg*_)	0.82 [0.74–0.90]	0.80 [0.72–0.88]	**0.81 [0.78–0.84]**

## Appendix A. Supplementary data

Supplementary data to this article can be found online at https://doi.org/10.1016/j.ejrad.2026.112776.
