# Supplementary material for: Image registration improves inter-reader agreement of objective response in CT assessment of pancreas adenocarcinoma
Source: Eur J Radiol. Author manuscript; Available in PMC 2026 Jun 8. (PMC13246015; doi:10.1016/j.ejrad.2026.112776)
Supplement: 1 [file NIHMS2179594-supplement-1.docx]

SUPPLEMENTAL MATERIAL

Contents

[Section S1: Segmentation Time 2](#_Toc224231064)

[Section S2: Impact of CT Tumor Density 3](#_Toc224231065)

[Supplemental Figure S1: Impact of CT Tumor Density 4](#_Toc224231066)

[Section S3: Agreement with Deep Learning Pancreas Segmentation 5](#_Toc224231067)

[Supplemental Table S1: Comparison of Deep Learning Pancreas Segmentation Methods 8](#_Toc224231068)

[Supplemental Table S2: Reader Confusion across Response Category 9](#_Toc224231069)

[Supplemental Table S3: Numerical Ranges of Survival Concordance Index 10](#_Toc224231070)

Section S1: Segmentation Time

The total segmentation and annotation time per image for junior readers was 16±3 minutes in pre-NAT images and 15±3 minutes in post-NAT images, whereas for senior readers the total time was 10±2 minutes in pre-NAT images and 9±2 minutes in post-NAT images. Total segmentation and annotation times were shorter among senior readers than among junior readers at baseline (*p*<0.001) and restaging (*p*<0.001). Paired analysis revealed that junior readers required more time to segment pre-NAT images than post-NAT images (*p*=0.006), whereas senior readers did not exhibit a difference in segmentation time between imaging timepoints (*p*=0.25). Considering that the total time required by junior readers compared to senior readers differed by approximately 50%, these findings suggest that experience level has a substantial impact on proficiency in longitudinal radiographic assessment of PDAC.

Section S2: Impact of CT Tumor Density

CT tumor density was measured by comparing the median intensity of the pancreatic background to the median intensity within the consensus tumor segmentation across all readers to. Hypodense PDAC were defined as tumors with <0 HU difference between the lesion and surrounding parenchyma, whereas isodense PDAC were defined as all non-hypodense lesions with ≥0 HU difference. At baseline, 24/30 (80%) lesions were hypodense and 6/30 (20%) were isodense. At restaging, 18/30 (60%) were hypodense and 12/30 (40%) were isodense. Through the course of NAT, 8/30 (27%) patients exhibited change in CT tumor density category, with an increase from hypodense to isodense in n=7 patients, and a decrease from isodense to hypodense in n=1 patient. Supplemental Figure S1 shows the GCI across all readers according to each category of CT tumor density. The GCI for isodense lesions was lower than the GCI for hypodense lesions (0.51 vs. 0.33, *p*<0.001, Wilcoxon rank sum test), suggesting that inter-reader variabilities are accentuated in isodense PDAC that tend to exhibit lower conspicuity. However, the modest reader agreements observed also imply that substantial measurement variabilities nonetheless persist in hypodense PDAC.

Supplemental Figure S1: Impact of CT Tumor Density


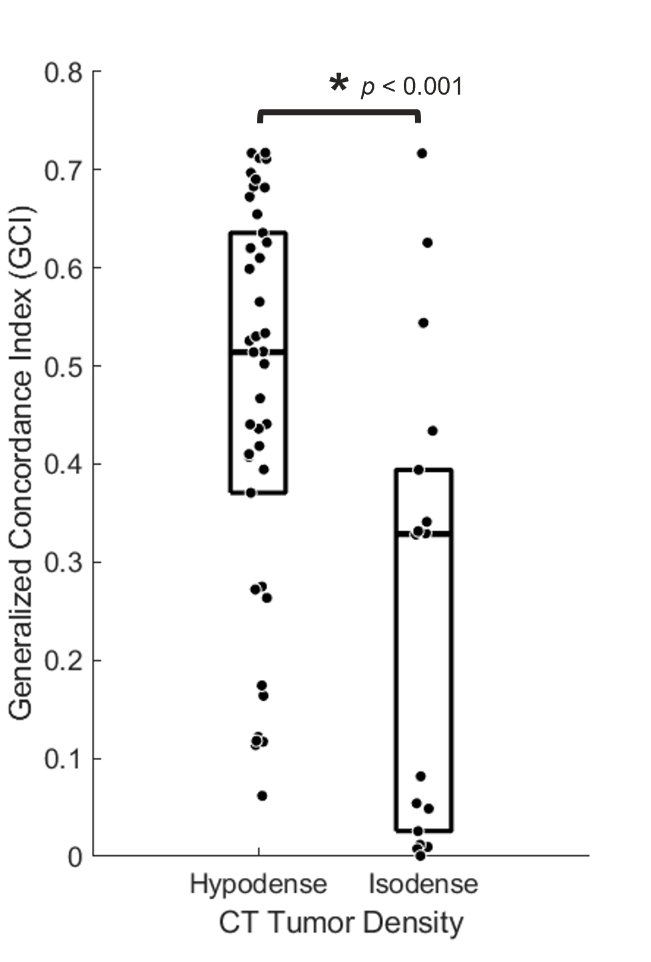


**Supplemental Figure S1.** Generalized concordance index (GCI) stratified by CT tumor density. Isodense PDAC exhibited lower GCI than hypodense PDAC.

Section S3: Agreement with Deep Learning Pancreas Segmentation

**Deep Learning Architecture**

To assess reliability of deep learning algorithms to accelerate pancreas segmentation, baseline and restaging CT images were processed using two pretrained deep learning segmentation approaches built on nnUNet architecture^[23]^. nnUNet is a state-of-the-art architecture that performs automatic configuration of image preprocessing, network size, data augmentation, training parameters, and post-processing steps associated with the segmentation task to enable high quality medical image segmentations across a variety of different datasets. The first segmentation algorithm was an in-house nnUNet model trained on institutional portal venous contrast-enhanced CT scans of PDAC patients. A total of 1100 scans were used to fine-tune the pre-trained nnUNet network^[23]^ for pancreas segmentation. The pancreas segmentations were manually performed by clinical fellows with guidance by experienced radiologists. The second algorithm was TotalSegmentator, a multi-organ segmentation network also based on an nnUNet backbone trained on 1082 annotated CT images of mostly healthy pancreases^[24]^. Neither deep learning algorithm was trained to perform PDAC tumor segmentation due to the lack of large datasets with high-quality PDAC lesion annotations. Both nnUNet networks were evaluated so that a comparison could be made between in-house software trained on CT pancreas containing tumor pathology and TotalSegmentator trained on normal pancreas.

**Variability in Pancreas Segmentation**

GCI of deep learning pancreas segmentations algorithms did not differ from GCI of senior readers in either pre-NAT (*p*=0.99) or post-NAT (*p*=0.73) images, suggesting an equivocal level of variability associated with the segmentation task between the deep learning approaches and expert radiologist readers. Supplemental Table S1 summarizes the agreements of the deep learning segmentation algorithms according to GCI (Jaccard index) and Dice overlap metrics with the segmentations generated by senior readers and the median consensus segmentation representing the average of all junior and senior reader segmentations. Mean Dice scores for the in-house nnUNet ranged from 0.71-0.82 while those for TotalSegmentator ranged from 0.79-0.87, suggesting better absolute agreement between TotalSegmentator and the senior and consensus segmentations than the in-house nnUNet (*p*<0.001).

Volumetric agreements of deep learning pancreas segmentations were strong, with CCC of 0.79 [0.63 - 0.88] for $V_{Pancreas}^{Pre}$, 0.86 [0.72 - 0.93] for $V_{Pancreas}^{Post}$, and 0.86 [0.72 - 0.93] for ${\%\Delta V}_{Pancreas}$. No differences in volumetric agreements were detected between automatic deep learning segmentation methods and senior readers (*p*=0.18, *p*=0.27, and *p*=0.71 for $V_{Pancreas}^{Pre}$, $V_{Pancreas}^{Post}$, and ${\%\Delta V}_{Pancreas}$, respectively).

**Relevance**

More broadly, high inter-reader variabilities associated with annotation-based assessments of PDAC tumor response suggest that additional developments are needed to improve precision and personalization of therapy. While improvements to imaging technologies and integration with non-imaging biomarkers^[35]^ may help to reduce uncertainties in assessment of therapeutic response, it is likely that medical image analysis and machine learning will lead to novel imaging markers for response assessment. However, the role of machine learning approaches in clinical practice is lacking due to reliance on manual segmentations, sensitivity to inter-reader variabilities, and limitations with generalizability^[14]^. As data-driven approaches continue to develop, it is likely that these methods will become increasingly potentiated by state-of-the-art deep learning algorithms. We find that deep learning segmentation algorithms for the whole pancreas operate with similar spatial agreements and volumetric variability to manual segmentation by senior radiologists. This consistency of deep learning algorithms approaching parity with expert performance on the whole organ pancreas segmentation task holds promise for automated assistance of radiologist workflows. However, the ability to accurately train deep learning models to accelerate PDAC tumor segmentation remains a practical barrier. More work towards improving workflow integration of computational assistance and surveillance of imaging markers within radiologists’ assessment and clinical interpretation of therapeutic response remains necessary to advancing AI-assisted capabilities for informing outcomes, stratifying patients, and personalizing PDAC treatment course.

Supplemental Table S1: Comparison of Deep Learning Pancreas Segmentation Methods

|  |  | **GCI** | |  | **Dice** | | **Dice**  ***p*-value** |
| --- | --- | --- | --- | --- | --- | --- | --- |
|  |  | **nnUNet** | **TotalSegmentator** |  | **nnUNet** | **TotalSegmentator** |  |
| Pre Pancreas | Senior 1 | 0.60 ± 0.07 | 0.69 ± 0.05 |  | 0.75 ± 0.06 | 0.81 ± 0.03 | <0.001 |
|  | Senior 2 | 0.67 ± 0.07 | 0.73 ± 0.05 |  | 0.80 ± 0.06 | 0.84 ± 0.03 | <0.001 |
|  | **Consensus** | **0.69 ± 0.07** | **0.77 ± 0.05** |  | **0.82 ± 0.06** | **0.87 ± 0.03** | <0.001 |
| Post Pancreas | Senior 1 | 0.55 ± 0.11 | 0.66 ± 0.06 |  | 0.71 ± 0.09 | 0.79 ± 0.05 | <0.001 |
|  | Senior 2 | 0.62 ± 0.11 | 0.71 ± 0.07 |  | 0.76 ± 0.09 | 0.83 ± 0.05 | 0.003 |
|  | **Consensus** | **0.64 ± 0.12** | **0.74 ± 0.07** |  | **0.77 ± 0.09** | **0.85 ± 0.05** | **0.001** |

**Supplemental Table S1.** Comparison of the in-house nnUNet and TotalSegmentator deep learning pancreas segmentation methods to senior radiologist segmentations and consensus segmentation of all (junior and senior) radiologist readers via generalized conformity index (GCI) and Dice score.

Supplemental Table S2: Reader Confusion across Response Category

|  |  | **Response Category** | | | |  | **Confusion Rate** | | |
| --- | --- | --- | --- | --- | --- | --- | --- | --- | --- |
|  |  | **PD** | **SD** | **PR** | **CR** |  | **PD/SD** | **SD/PR** | **Total** |
| RECIST v1.1 ($\%\Delta SLD$) | Junior 1 | 4 (13%) | 21 (70%) | 5 (17%) | 0 (0%) |  | 4 (13%) | 8 (27%) | 12 (40%) |
|  | Junior 2 | 2 (7%) | 25 (83%) | 3 (10%) | 0 (0%) |  |  |  |  |
|  | Senior 1 | 1 (3%) | 25 (83%) | 4 (13%) | 0 (0%) |  | 0 (0%) | 5 (17%) | 5 (17%) |
|  | Senior 2 | 1 (3%) | 26 (87%) | 3 (10%) | 0 (0%) |  |  |  |  |
|  | **All Readers** | **1 (3%)** | **25 (83%)** | **4 (13%)** | **0 (0%)** |  | **4 (13%)** | **10 (33%)** | **14 (47%)** |
| WHO Criteria ($\%\Delta SDP$) | Junior 1 | 4 (13%) | 20 (67%) | 6 (20%) | 0 (0%) |  | 4 (13%) | 8 (27%) | 12 (40%) |
|  | Junior 2 | 2 (7%) | 26 (87%) | 2 (7%) | 0 (0%) |  |  |  |  |
|  | Senior 1 | 1 (3%) | 25 (83%) | 4 (13%) | 0 (0%) |  | 1 (3%) | 5 (17%) | 6 (20%) |
|  | Senior 2 | 2 (7%) | 25 (83%) | 3 (10%) | 0 (0%) |  |  |  |  |
|  | **All Readers** | **1 (3%)** | **26 (87%)** | **3 (10%)** | **0 (0%)** |  | **5 (17%)** | **11 (37%)** | **16 (53%)** |
| RECIST v1.1 (${\%\Delta V}_{Seg}$) | Junior 1 | 1 (3%) | 10 (33%) | 19 (63%) | 0 (0%) |  | 0 (0%) | 18 (60%) | 18 (60%) |
|  | Junior 2 | 1 (3%) | 14 (47%) | 15 (50%) | 0 (0%) |  |  |  |  |
|  | Senior 1 | 1 (3%) | 14 (47%) | 15 (50%) | 0 (0%) |  | 1 (3%) | 8 (27%) | 9 (30%) |
|  | Senior 2 | 3 (10%) | 15 (50%) | 13 (43%) | 0 (0%) |  |  |  |  |
|  | **All Readers** | **1 (3%)** | **10 (33%)** | **19 (63%)** | **0 (0%)** |  | **1 (3%)** | **21 (70%)** | **22 (73%)** |
| RECIST v1.1 (${\%\Delta V}_{Reg}$) | Junior 1 | 1 (3%) | 23 (77%) | 6 (20%) | 0 (0%) |  | 0 (0%) | 2 (7%) | 2 (7%) |
|  | Junior 2 | 1 (3%) | 23 (77%) | 6 (20%) | 0 (0%) |  |  |  |  |
|  | Senior 1 | 1 (3%) | 24 (80%) | 5 (17%) | 0 (0%) |  | 1 (3%) | 1 (3%) | 2 (7%) |
|  | Senior 2 | 2 (7%) | 24 (80%) | 4 (13%) | 0 (0%) |  |  |  |  |
|  | **All Readers** | **1 (3%)** | **24 (80%)** | **5 (17%)** | **0 (0%)** |  | **1 (3%)** | **3 (10%)** | **4 (13%)** |

**Supplemental Table S2.** Number of patients stratified into each response category indexed according to criterion scores as evaluated by independent readers. Rows summarizing across all readers reflect the consensus based on the predominant category. Confusion rates across categories are reported as number of patients with discordant reader categorizations. PD: progressive disease; SD: stable disease; PR: partial response; CR: complete response.

Supplemental Table S3: Numerical Ranges of Survival Concordance Index

| **Quantity** | **C-index, OS** | **C-index, RFS** |
| --- | --- | --- |
|  | *Annotation* |  |
| $\%\Delta SLD$ | 0.50 ± 0.07 [0.36 - 0.64] | 0.50 ± 0.08 [0.34 - 0.67] |
| $\%\Delta SDP$ | 0.50 ± 0.07 [0.36 - 0.64] | 0.49 ± 0.08 [0.33 - 0.64] |
|  | *Segmentation* |  |
| ${\%\Delta V}_{Seg}$ | 0.55 ± 0.07 [0.42 - 0.68] | 0.54 ± 0.07 [0.41 - 0.67] |
| ${\Delta\%B}_{Seg}$ | 0.61 ± 0.06 [0.49 - 0.73] | 0.60 ± 0.07 [0.46 - 0.74] |
|  | *Registration* |  |
| ${\%\Delta V}_{Reg}$ | 0.58 ± 0.07 [0.44 - 0.72] | 0.51 ± 0.08 [0.36 - 0.66] |
| ${\Delta\%B}_{Reg}$ | 0.63 ± 0.06 [0.52 - 0.74] | 0.54 ± 0.08 [0.38 - 0.70] |

**Supplemental Table S3.** Harrell’s C-index with 95% confidence intervals for agreement of annotation-, segmentation-, and registration-based measures of change in tumor size across NAT. SLD: sum of longest diameters; SDP: sum of diameter products; ${\%\Delta V}_{Seg}$: percent change in tumor volume via image segmentation; ${\%\Delta B}_{Seg}$: change in tumor burden ratio via image segmentation; ${\%\Delta V}_{Reg}$: percent change in tumor volume via image registration; ${\%\Delta B}_{Reg}$: change in tumor burden ratio via image registration.
